# Preparation of nitrogen-doped porous carbons for high-performance supercapacitor using biomass of waste lotus stems†

**DOI:** 10.1039/c7ra13013a

**Published:** 2018-02-12

**Authors:** Song Yan, Jingjing Lin, Ping Liu, Zhicheng Zhao, Jun Lian, Wei Chang, Lu Yao, Yueran Liu, Hualin Lin, Sheng Han

**Affiliations:** School of Chemical and Environmental Engineering, Shanghai Institute of Technology Haiquan Road 100 201418 Shanghai P. R. China hansheng654321@sina.com lhl6534@163.com +86-021-60873560 +86-021-60873228 +86-13524694909 +86-17 701878558

## Abstract

In this study, advanced nitrogen-doped porous carbon materials for supercapacitor was prepared using low-cost and environmentally friendly waste lotus stems (denoted as LS-NCs). Nitrogen in the surface functionalities of LS-NCs was investigated using X-ray photoelectron spectroscopy analysis. The sum of pyridine nitrogen (N-6) and pyrrolic/pyridinic (N-5) contents accounted for 94.7% of the total nitrogen and significantly contributed to conductivity. Pore structure and surface area of activated carbons were measured using the Brunauer–Emmett–Teller method. A maximum specific surface area of 1322 m^2^ g^−1^ was achieved for LS-NCs. The porous carbons exhibited excellent electrochemical properties with a specific capacitance of 360.5 F g^−1^ at a current density of 0.5 A g^−1^ and excellent cycling stability (96% specific capacitance retention after 5000 cycles). The above findings indicate that taking advantage of the unique structure of abundant waste lotus stem provides a low-cost and feasible design for high-performance supercapacitors.

## Abstract 2

In this study, advanced nitrogen-doped porous carbon materials for supercapacitor was prepared using low-cost and environmentally friendly waste lotus stems (denoted as LS-NCs).

## Graphical abstract

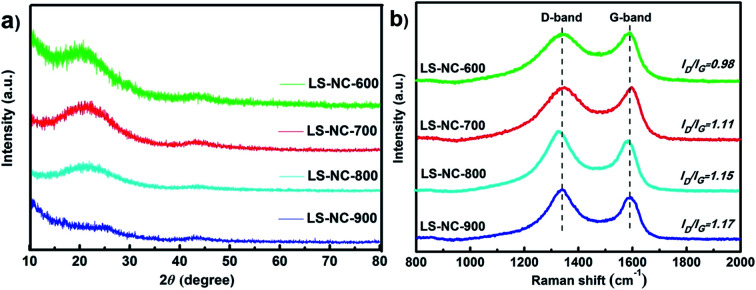

## 1. Introduction

Supercapacitors are drawing much attention as promising energy storage devices owing to their long cycle life, high power density, high chemical stability and low maintenance cost.^1–4^ Electrode materials are important determinants of supercapacitor performance.^5,6^ Nowadays, porous carbon materials have been widely used as electrode material of supercapacitors because of their high surface area and good electrical conductivity.^7^ Different methods and carbon sources are used to prepare porous carbon materials, which possess various pore sizes and structures. Selecting appropriate preparation methods and carbon precursors bears importance in adjusting pore structure parameters and lowering the cost of porous carbons.^8,9^ However, traditional carbon precursor materials are expensive and can cause serious impact on the environment, enormously hindering practical application of carbon-based materials in supercapacitors.^10^ Thus, the study for an inexpensive, environmental friendly and advanced electrode material has become a hot topic in the field of renewable energy research.

In recent years, bio-derived activated carbon materials have been widely investigated because of their good conductivity and low cost.^11,12^ As a renewable energy resource, biomass can be utilised for preparing porous carbon materials; not only can it reduce the cost of manufacturing, but it also improves waste recycling and development. For example, coconut shells are broadly used in production of commercial activated carbons.^13,14^ More natural biomass materials, such as leaves,^15^ pistachio nutshells,^16^*auricularia*,^17^ shiitake mushroom,^18^ longan shells,^19^ potato,^20^ waste celtuce leaves^21^ and cherry stone^22^ are also used in production of commercial activated carbons. As a perennial aquatic herb, lotus stems possess several large pores and abundant longitudinal ventilation holes, and its microstructure benefits improvement of electrochemical performance of samples. Lotus stems are widely distributed in China; some of them are used for traditional Chinese medicine, but the remaining majority are directly abandoned in rural areas, causing environmental pollution and waste of resources. In addition, discarded lotus stem waste draff is rich in cellulose and can be used to prepare carbon materials; such application opens an effective avenue for utilising discarded lotus stem waste draff as resources.

**Scheme 1:**
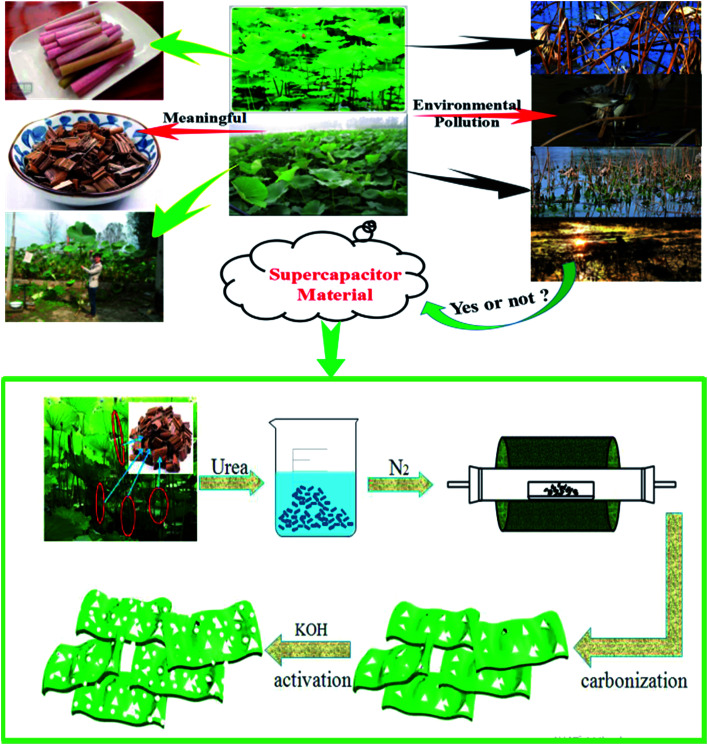
The schematic diagram for the synthesis of LS-NCs.

For this research, waste lotus stems were treated as carbon sources to prepare porous carbon by pyrolysis at 600 °C in nitrogen and followed by KOH activation to improve specific area of the material. Urea contains abundant nitrogen, which can improve electronic conductivity of carbon materials. The tube and lamellar of lotus stems provide an excellent platform to further optimise its structure and property and can be directly utilised as high-performance supercapacitors. Synthesis procedures of LS-NCs are shown in Scheme 1.

## 2. Experimental section

### 2.1. Material

Concentrated hydrochloric acid (HCl, 36–38%, wt) was purchased from Adamas-beta Inc., Shanghai, China. Urea and KOH were purchased from Sinopharm Chemical Reagent Co., Ltd. All aqueous solutions were prepared with deionised (DI) water. All chemicals were of analytical grade and were used without further purification.

### 2.2. Synthesis of activated lotus stems

Nitrogen-doped porous carbon was prepared using abandoned lotus stems as carbon source, KOH as activator and urea as nitrogen source. Typical carbonisation and activation processes are as follows. Lotus stem waste materials were collected from the river and cut into small pieces, washed with DI water for several times, dried at 100 °C for 24 h and then ground to powder. The powdered lotus stems were immersed in urea solution for 4 h, where urea and lotus stems were mixed at 3 : 1 weight ratio. The solution was dried at 60 °C for 12 h. Finally, lotus stems were carbonised under N_2_ atmosphere at 500 °C for 2 h and heating rate of 5 °C min^−1^.

Carbonised lotus stems were chemically activated. Carbonised lotus stems (1 g) and KOH (2 g) were added to ethanol solution (50 wt%, 10 ml), impregnated for 4 h and dried in an oven at 60 °C for 12 h. The dried mixture was activated under N_2_ atmosphere at 600 °C, 700 °C, 800 °C and 900 °C for 2 h. After activation, the obtained carbon materials were washed with aqueous HCl solution (2 M) to remove any inorganic salts, then rinsed with DI water and ethanol for several times until a pH of 7 was reached and dried at 60 °C for 3 h. The activated nitrogen-doped porous carbon is denoted as LS-NC-*X* in further discussions, where *X* represents activation temperature (600 °C, 700 °C, 800 °C and 900 °C).

### 2.3. Material characterisation

Powder X-ray diffraction (XRD) patterns were collected with a Bruker Focus *D*_8_ diffractometer with Cu Kα radiation (40 kV, 40 mA, *k* = 1.5418 Å) from 10° to 80°. Brunauer–Emmett–Teller (BET) method was utilised to assess specific surface areas and pore size by using a surface and pore size analysis instrument (3H-2000PM, Beishide Instrument-S & T Co., Ltd., China) at 77.3 K. X-ray photoelectron spectroscopy (XPS, AXIS Ultra DLD system from Kratos) spectra were derived from a Specs spectrometer by using Al Kα radiation as excitation source. Structure and morphology of porous carbons were obtained using transmission electron microscopy (TEM, JEM-2100, JEOL) and field-emission scanning electron microscopy (Hitachi S-4800). Raman spectra were collected on a LabRAM HR800 Raman spectrometer. Weight loss behaviour of lotus stem was measured using a thermogravimetric analyser (DTG-60AH), as shown in Fig. S1.†

### 2.4. Electrochemical measurement

Electrochemical behaviour of LS-NC-X was measured using cyclic voltammetry (CV), galvanostatic charge–discharge (GCD) and electrochemical impedance spectroscopy (EIS); measurements were conducted on a CHI 760E electrochemical workstation. Cycle stability was determined using a CT2001A Land testing equipment. The working electrode was prepared using active materials (LS-NC-*X*), acetylene carbon black and polytetrafluoroethylene at a mass ratio of 8 : 1 : 1. A total of 1.0 ml anhydrous ethanol was mixed with active materials to prepare a homogeneous slurry. Approximately 2 mg of active material was coated on foamed nickel sheet and dried overnight at 60 °C. The employed three-electrode and two-electrode configuration are consisted of electrode materials with the size of (1.0 cm × 1.0 cm), and nickel foam as the current collectors. All electrochemical tests were performed using a standard three-electrode and two-electrode system with a Hg/HgO electrode as reference electrode and platinum electrode as counter electrode in an aqueous electrolyte solution (6 M KOH) at 25 °C.

Specific capacitance of the electrode was calculated based on GCD measurements and according to the following equation:^23^*C*_*m*_ = *I*Δ*t*/*m*(Δ*V*)where *I* (A), Δ*t* (s), *m* (g) and Δ*V* (V) refer to discharge current, discharge time, mass of the active material and voltage window, respectively.

## 3. Results and discussions

### 3.1. Characterisation of structure and morphology

**Fig. 1:**
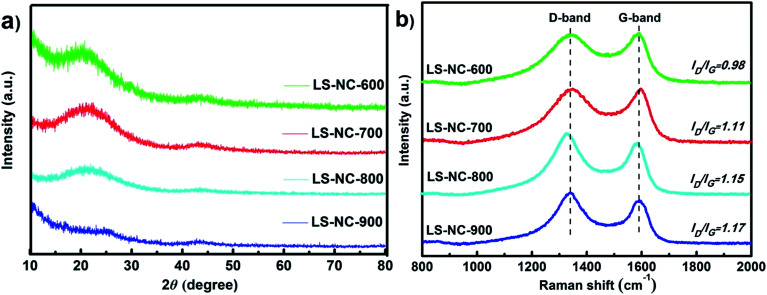
(a) XRD patterns and (b) Raman spectra of LS-NC-600, LS-NC-700, LS-NC-800 and LS-NC-900, respectively.

Crystallite structure and characteristics of LS-NCs were investigated using XRD and Raman spectrometry analyses. As shown in Fig. 1a, LS-NCs exhibited two broad peaks at 2*θ* diffraction angles of 23° and 43°, which correspond to (002) and (100) reflection of turbostratic graphite, respectively.^24,25^ LS-NC-600 showed an additional peak at 11°, indicating more oxygen functional groups in the sample.^26^ Peak intensity at 22° decreased with improvement of activation temperature; this result indicates that LS-NC structure was seriously destroyed by activation of KOH at elevated temperatures.^27^

Raman spectroscopy was utilised to further illuminate LS-NCs structures, and results are shown in Fig. 1b. Two distinctive peaks can be observed in all specimens, and they correspond to D (1350 cm^−1^) and G (1590 cm^−1^) band. Integral ratio (*I*_D_/*I*_G_) is estimated to indicate degree of structural disorder with respect to a perfect graphitic structure.^28^ In the present study, ratios of LS-NC-600, LS-NC-700, LS-NC-800 and LS-NC-900 reached 0.98, 1.11, 1.15 and 1.17, respectively. With increasing activation temperature, *I*_D_/*I*_G_ ratios gradually increased, indicating that order of graphitic structure was destroyed, and that deeper activation has promoted the presence of defects in LS-NCs.^29^ The above XRD pattern and Raman spectrum results imply that these LS-NCs are multiaperture-activated carbon material with disordered carbon structure.

**Fig. 2:**
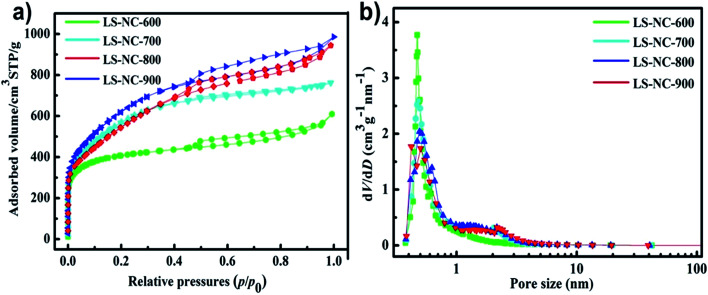
(a) BET nitrogen adsorption–desorption isotherm of LS-NC-600, LS-NC-700, LS-NC-800 and LS-NC-900, respectively. (b) Corresponding pore size of LS-NC-600, LS-NC-700, LS-NC-800 and LS-NC-900, respectively.

N_2_ adsorption–desorption isotherms of LS-NCs were measured at −196 °C to investigate the surface area and porous structure of LS-NCs, as shown in Fig. 2a and Table. S1.† LS-NCs showed typical IV nitrogen adsorption–desorption isotherms curves, indicating the existence of micropores and mesopores.^30,31^ Isotherms of the obtained LS-NCs exhibited a relatively broad keen in the low-pressure range, implying the existence of microporous structure. LS-NCs possessed a shot-range hysteresis loop at relative pressure *P*/*P*_0_ from 0.45 to 0.90, exhibiting a well-ordered mesoporous structure.^32^ Table. S1† summarises porous textural details of these LS-NCs materials. The pore size distribution shown in Fig. 2b was determined through density functional theory. For all LS-NCs, the peaks that centred at about 0.5 nm indicate that LS-NCs possessed micropore and mesopore structures. Along with rising activation temperature from 600 °C to 900 °C, specific surface areas of LS-NCs reached 1322, 2013, 2221 and 1986 m^2^ g^−1^ (Table. S1†). Specific surface area of the material first increased and then decreased with increasing activation temperature; this result can be attributed the excessive temperature, resulting in enhancement of KOH activation and collapse of the structure. Furthermore, LS-NC-800 yielded high BET surface area and micropore volume. This hierarchical porous structure with high surface area plays a key role in enhancing ion transport and charge storage.

**Fig. 3:**
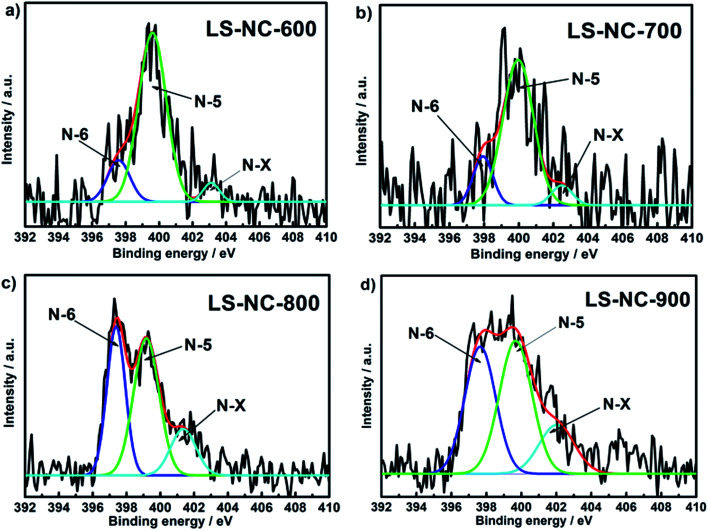
N 1s spectra of the nitrogen-doped porous carbons (a) LS-NC-600; (b) LS-NC-700; (c) LS-NC-800; (d) LS-NC-900, respectively.

The nature of nitrogen on surface functionalities of LS-NCs was further investigated using XPS analysis (Fig. 3). As shown in Fig. S2,† the coexistence of C, N and O was confirmed by XPS survey, suggesting that nitrogen was successfully doped into porous carbons. N 1s core level spectra of the LS-NCs are exhibited in Fig. 3 and Table 1. As predicted, the samples featured three peaks at 397.6, 399.6 and 403.2 eV, referring to the three types of nitrogen species, namely, pyridine nitrogen (N-6), pyrrolic/pyridinic nitrogen (N-5) and oxidised nitrogen (N-X), respectively. N-6 percentage increased from 39.1% to 79.1%, whereas N-5 percentage decreased from 43.3% to 15.6% in LS-NC-900, LS-NC-800, LS-NC-700 and LS-NC-600. A careful observation showed that by comparing with the results of LS-NC-600, LS-NC-700 and LS-NC-800, LS-NC-900 summarized in Table 1. The total nitrogen contents in LS-NCs were found to be inversely proportional to the activation temperature, indicating that higher temperature will lead to more loss of nitrogen atoms during activation, this trend is accordance with the reports elsewhere.^33,34^ In many previous reports, N-6 and N-5 located at the edges of grapheme layers are considered representing the pseudo capacitive effect in aqueous electrolyte, which are of importance to improve the capacitance characteristics for nitrogen-doped carbon materials.^35,36^ In general, the N-6 and N-5 in the carbons were regarded as electroactive sites, which would benefit the enhancement of electrical conductivity as well as capacitances.^37^ These results illustrate increasing total contents of N-6 and N-5, similar to previous results indicating that high N-6 and N-5 contents in porous carbons significantly contribute to conductivity.^38^

**Table 1:** Percentage of various species of nitrogen in the total nitrogen of the LS-NCs

Sample	Nitrogen content (at%)		
	N-6 (397.6 eV)	N-5 (399.6 eV)	N-Q (403.2 eV)
LS-NC-600	79.1	15.6	5.3
LS-NC-700	76.3	16.2	7.5
LS-NC-800	47.2	36.4	16.4
LS-NC-900	39.1	43.3	17.6

**Fig. 4:**
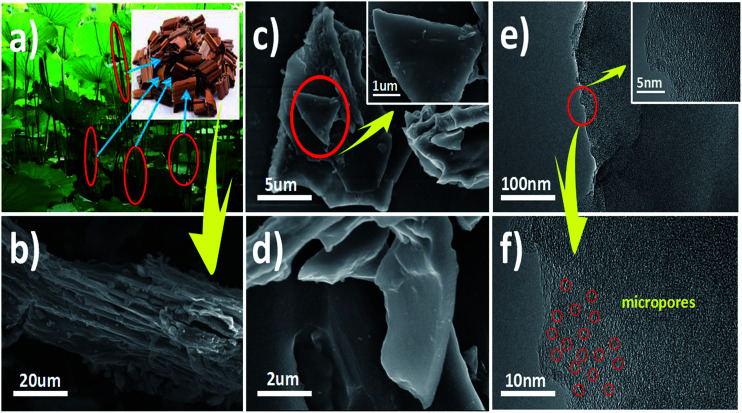
(a) The photographic images of lotus stem; (b) the SEM images of lotus stem; (c, d) the SEM images of LS-NC-600; (e, f) the SEM images of LS-NC-600.

Fig. 4a and b present photographic images and SEM images of lotus stems. The lotus stems presented numerous lamellar and thin-slice appearance, contributing to molten KOH permeation for activation. As shown in Fig. 4c and d, numerous microporous structures were observed when temperature was increased to 600 °C. TEM images of pore structure are presented in Fig. 4e and f. These two TEM images confirm that LS-NC-600 comprises turbostratic carbon with a disordered graphitic microstructure. This unique microporous structure benefits improvement of material conductivity. SEM and mapping images of LS-NC-600 are shown in Fig. S3,† which also displays uniform distribution of C, O and N on the surface. Nitrogen content is relatively small, corresponding to XPS results.

### 3.2. Electrochemical performance

**Fig. 5:**
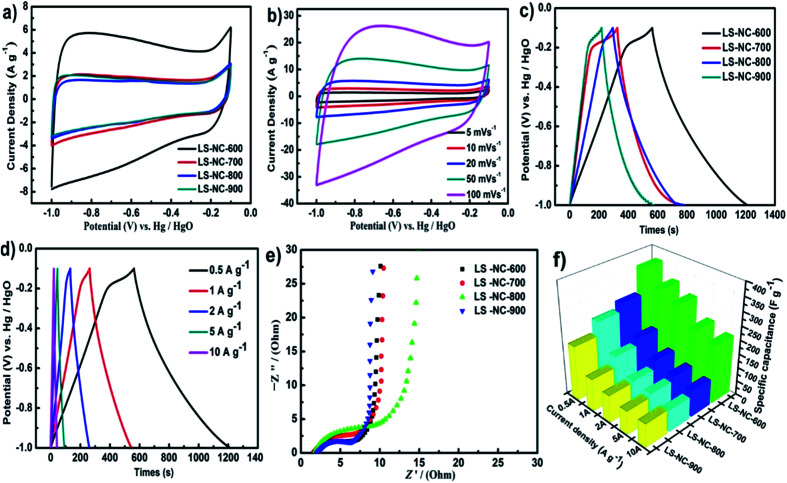
(a) CV curves of different electrodes at a scan rate of 20 mV s^−1^ in 6 M KOH aqueous solution; (b) CV curves of LS-NC-600 at different scan rates; (c) GCD curves at a current density of 0.5 A g^−1^; (d) GCD curves of LS-NC-600 at different current density; (e) Nyquist plots base on LS-NC-600 electrodes; (f) specific capacitance at current densities at different current densities in the range of 0.5–10 A g^−1^.

Electrochemical performance of LS-NCs was investigated using CV, GCD and EIS analysis in 6 M KOH electrolyte by a three-electrode system to explore potential applications of LS-NCs as electrode materials. Fig. 5a displays CV curves of LS-NC-600, LS-NC-700, LS-NC-800 and LS-NC-900 electrode materials at a scan rate of 20 mV s^−1^. All samples displayed a typical quasirectangular shape, indicating the ideal capacitance of the double layer. In comparison with the CV curves of LS-NC-700, LS-NC-800 and LS-NC-900, the curve of LS-NC-600 exhibited a larger rectangular sharp, suggesting the excellent electrochemical performance of LS-NC-600; this result can be attributed to the microporous structure and high N-5 and N-6 contents. Fig. 5b illustrates the CV curves of LS-NC-600 at different scan rates from 5 mV s^−1^ to 100 mV s^−1^. For an ideal double-layer capacitor, the CV curves exhibited perfect rectangular-shaped profile. With decreasing scan rates from 100 mV s^−1^ to 5 mV s^−1^, the CV curves showed similar and perfect rectangular cross sections, indicating the excellent capacitive property of LS-NC-600.

GCD test was also conducted to explore capacitance performance of four LS-NCs at a current density of 0.5 A g^−1^, as shown in Fig. 5c. Fig. 5d presents the GCD curves of LS-NC-600 at various current densities from 2 A g^−1^ to 10 A g^−1^. LS-NC-600 electrode manifested the longest discharge time with specific capacitance as high as 360.5 F g^−1^ at 0.5 A g^−1^. Specific capacitance values of LS-NC-700, LS-NC-800 and LS-NC-900 reached 269.1, 243.3 and 195.1 F g^−1^, respectively. The high specific capacitance of LS-NC-600 may be attributed to its well-developed porous structure and abundant N-5 and N-6 contents, which can improve charge transmission capacity and further promote electrochemical capacitive properties. Although the specific surface area of LS-NC-600 was not the highest, its specific capacitance was the highest (360.5 F g^−1^ at 0.5 A g^−1^); this result shows that N-5 and N-6 can enhance electrochemical properties of materials. LS-NC-600 featured better electrochemical performance than some biomass carbon materials mentioned in previous literature (Table S2†). Fig. 5a, the CV curves show obviously polarization peak at the voltage about −0.2 V, and from Fig. 5b and c, GCD curves do not exhibit a symmetrical shape as stated. Basically, these due to the following factor: at higher scan rates, the larger the ion transport resistance in the pores, which impedes the formation of the electrical double layer.^39^ This phenomenon indicates an excellent pseudo-capacitance effect through the nitrogen functional group.^40,41^

As a contrast, Fig. S4a† shows the CV curves of LS-NC-500 at different scan rates. The CV coves of LS-NC-500 at different scan rates display a typical electric double-layer capacitor characteristic.^42^ When the scan rate is increased to 100 mV s^−1^, the CV curves is a little deviate the typical rectangular shape, which is due to that the larger ion transport resistance in the pores impedes the formation of the electrical double layer at higher scan rates.^43^ The GCD curves of LS-NC-500 at different current density are illustrated in Fig. S4b.† When current density increased from 0.5 A g^−1^ to 10 A g^−1^, the GCD curves of LS-NC-500 always remain in a nearly triangular shape. The specific capacitance values of LS-NC-500 is 186.5 F g^−1^ at 0.5 A g^−1^, which is lower than LS-NC-600, may be attributed to its undeveloped porous structure and lower nitrogen content synergistic effect.^44^ This results are correspond to the early study.

**Fig. 6:**
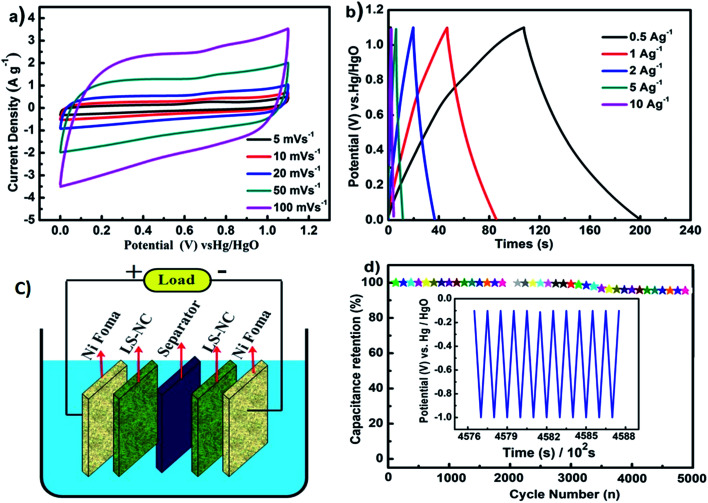
(a) CV curves of LS-NC-600 at different scan rates in 6 M KOH aqueous solution; (b) GCD curves of LS-NC-600 at different current density; (c) schematic of the LS-NC symmetric supercapacitor; (d) the cycling performance of the LS-NC-600 as electrode in three electrodes at the current density of 5 A g^−1^.

Nyquist plots of the LS-NCs are shown in Fig. 5e. The small semicircle in the high-frequency region represents charge-transfer resistance,^45^ which is controlled by reaction kinetics. LS-NC-600 exhibited an almost vertical line at low frequency, implying its potential as an ideal capacitor.^46^Fig. 5f summarises specific capacitance at different current densities in the range of 0.5–10 A g^−1^. With increasing current density, specific capacitance decreased, which can be attributed to the increase in diffusion limitation. In comparison with LS-NC-700, LS-NC-800 and LS-NC-900, LS-NC-600 electrode showed the highest specific capacitance, thereby implying its excellent rate capability. These results indicate the synergistic effects between nitrogen atoms doped in the electrode material and high specific surface area of the porous structure. Cycling stability is a key parameter for supercapacitor in practical applications. As shown in Fig. 6d, the LS-NC-600 electrode exhibited excellent stability with specific capacitance retention of 96% after 5000 cycles in three electrodes at the current density of 5 A g^−1^, suggesting its possible use for supercapacitor applications.

A two-electrode symmetric supercapacitor was assembled to evaluate energy storage performance of supercapacitors, as shown in the schematic in Fig. 6c. As shown in Fig. 6a, CVs of LS-NC-600 retained a rectangular shape as the scan rate was increased from 5 mV s^−1^ to 100 mV s^−1^, indicating rapid charge–discharge characteristics and good capacitance properties at high scan rates. All charge–discharge curves exhibited a typical double layer capacitance behaviour, as shown in Fig. 6b, which implies small internal resistance. High N content, hierarchical pore structure and good conductivity of the LS-NC-600 electrode led to low mass transfer resistance.

## 4. Conclusions

LS-NCs were prepared from a green, low-cost, abundant and sustainable biological resource, waste lotus stems, by subsequent KOH activation. High N-6 and N-5 contents in the porous carbon may improve conductivity. Nitrogen-doped porous carbon showed a maximum specific surface area of 1322 m^2^ g^−1^, extraordinary microporous structure and superior electrochemical performance. The as-prepared samples exhibited excellent supercapacitor properties with high specific capacitance of 360.5 F g^−1^ at 0.5 A g^−1^. The electrode displayed excellent stability with 96% capacitance retention after 5000 cycles, indicating the feasibility of utilising waste biomass to produce promising low-cost materials for high-performance commercial supercapacitors. These findings highlight the possible utilisation of waste biomass for other energy storage applications, such as Li-ion batteries and hydrogen storage.

## Conflicts of interest

There are no conflicts to declare.

## Supplementary Material

RA-008-C7RA13013A-s001
