# Supplementary material for: Preparation of nitrogen-doped porous carbons for high-performance supercapacitor using biomass of waste lotus stems
Source: RSC Adv. 2018 Feb 12;8(13):6806–13. doi: 10.1039/c7ra13013a (PMC9078325; doi:10.1039/c7ra13013a)
Supplement: RA-008-C7RA13013A-s001 [file RA-008-C7RA13013A-s001.pdf]

Electronic Supplementary Material (ESI) for RSC Advances.
This journal is © The Royal Society of Chemistry 2018

Supporting Information

Preparation of nitrogen-doped porous carbons with high-performance supercapacitor using biomass of waste lotus stems

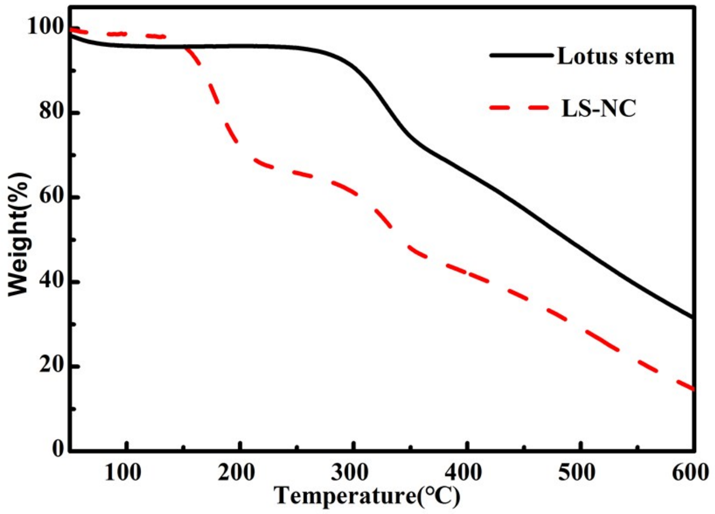

Fig. S1 TGA curves of waste Lotus stem and LS-NC under N_2 atmosphere.

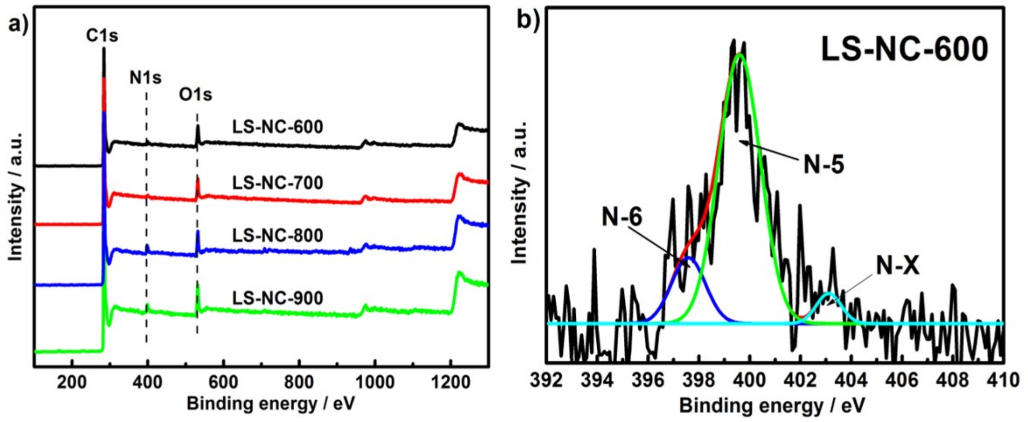

Fig. S2 General XPS spectrum of LS-NC-600, LS-NC-700, LS-NC-800 and LS-NC-900, respectively.

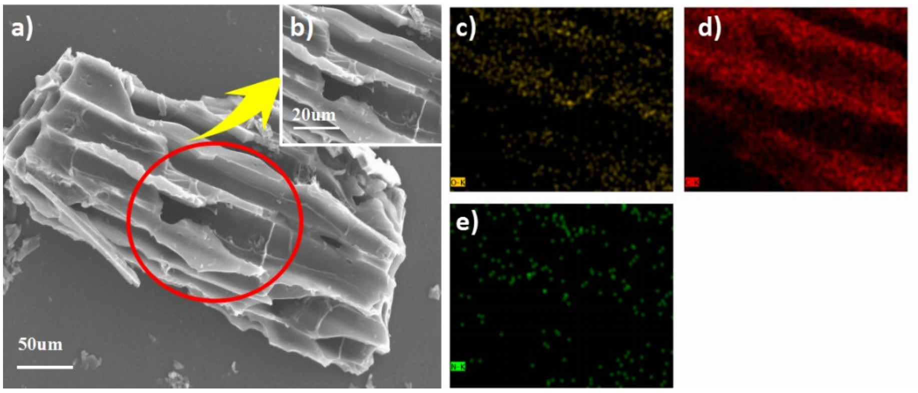

Fig. S3 The SEM images and mapping images of LS-NC-600.

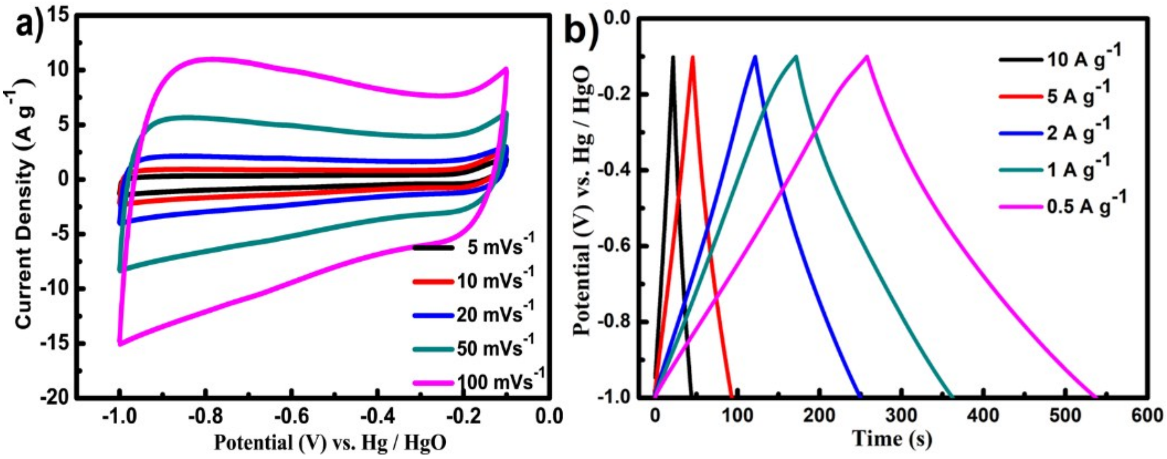

Fig. S4 a) CV curves of LS-NC-500 at different scan rates; b) GCD curves of LS-NC-500 at different current density

Table. S1 Textural parameters of LS-NC-600, LS-NC-700, LS-NC-800 and LS-NC-900, respectively.

Sample	Textural properties			
	$S_{BET}$$^{a)}$[$m^2g^{-1}$]	$S_{BET}$$^{b)}$[$m^2g^{-1}$]	$V_p$$^{d)}$[$m^3g^{-1}$]	$D_a$$^{d)}$[nm]
LS-NC-600	1322	1088	0.94	2.85
LS-NC-700	2013	1800	1.17	2.34
LS-NC-800	2221	1876	1.52	2.74
LS-NC-900	1986	1610	1.46	2.94

a) The specific surface areas were calculated using the BET method.
b) Micropore surface area calculated from t-plot method.
c) The total pore volume was determined from the amount of nitrogen adsorbed at a relative pressure of 0.99
d) Average pore diameter.

Table. S2 Summary of reported capacitive performance of the carbon materials.

Biomass precursors	C_m / F g^{-1}	Electrolyte	Reference
Algae	234 (at 0.5 A g^{-1})	6 M KOH	1
longan shell	322 (at 0.5 A g^{-1})	6 M KOH	2
Prawn shells	288 (at 5 A g^{-1})	6 M KOH	3
Enteromorpha prolifera	296 (at 0.5 A g^{-1})	30 wt% KOH	4
Willow catkin	298 (at 0.5 A g^{-1})	6 M KOH	5
Potato	255 (at 0.5 A g^{-1})	2 M KOH	6
Rice husk	243 (at 0.05 A g^{-1})	6 M KOH	7
Horseweed	184 (at 0.4 A g^{-1})	6 M KOH	8
**Lotus stem**	**360 (at 0.5 A g^{-1})**	**6 M KOH**	This work

References:

1 Z. Tian, M. Xiang, J. Zhou, L. Hu and J. Cai, *Electrochim. Acta.,* 2016, **211**, 225-233.

2 H. Wei, H. Chen, N. Fu, J. Chen, G. Lan, W. Qian, Y. Liu, H. Lin and S. Han, *Electrochim. Acta.,* 2017, **231**, 403-411.

3 F. Gao, J. Qu, Z. Zhao, Z. Wang and J. Qiu, *Electrochim. Acta.,* 2016, **190**, 1134-1141.

4 X. Gao, X. Wei, J. Zhou, G. Wang, S. Zhuo, Z. Liu, Q. Xue and Z. Yan, *Electrochim. Acta.,* 2014, **133**, 459-466.

5 Y. Li, G. Wang, T. Wei, Z. Fan and P. Yan, *Nano Energy.,* 2016, **19**, 165-175.

6 G. Ma, Q. Yang, K. Sun, H. Peng, F. Ran, X. Zhao and Z. Lei, *Bioresour. Technol.,* 2015, **197**, 137.

7 X. He, P. Ling, M. Yu, X. Wang, X. Zhang, M. Zheng, X. He, P. Ling, M. Yu and X. Wang, *Electrochim. Acta.,* 2013, **105**, 635-641.

8 L. Yuan, C. Feng, C. Wang, Z. Fu, X. Yang and Y. Tang, *J. Mater. Sci.,* 2016, **51**, 3880-3887.
